# A case of small intestinal fixation failure

**DOI:** 10.1093/bjrcr/uaae046

**Published:** 2024-11-27

**Authors:** Kanako Oyanagi, Yosuke Horii, Hiroyuki Ishikawa, Kazuyasu Takizawa

**Affiliations:** Department of Radiology and Radiation Oncology, Niigata University Graduate School of Medical and Dental Sciences, Niigata 951-8510, Japan; Department of Radiology and Radiation Oncology, Niigata University Graduate School of Medical and Dental Sciences, Niigata 951-8510, Japan; Department of Radiology and Radiation Oncology, Niigata University Graduate School of Medical and Dental Sciences, Niigata 951-8510, Japan; Division of Digestive and General Surgery, Niigata University Graduate School of Medical and Dental Sciences, Niigata 951-8510, Japan

**Keywords:** ligament of Treitz, duodenal-jejunal junction, anterior pararenal space

## Abstract

A 77-year-old woman underwent CT to evaluate haematemesis. The images showed that the third part of the duodenum flexed steeply on the right side of the aorta and ran caudally, without crossing anterior to the aorta. The duodenal-jejunal junction and jejunum were located on the patient’s right side. Upper gastrointestinal endoscopy revealed a laceration at the gastric cardia, and a diagnosis of Mallory–Weiss syndrome was made. Repeat CT 7 days later revealed that the abnormal positioning of the intestinal tract had resolved spontaneously. Two months later, the patient experienced another episode of haematemesis, and CT revealed repeat deviation of the duodenal-jejunal junction and jejunum to her right side. Upper gastrointestinal endoscopy revealed another laceration at the gastric cardia, as in the previous study. On the basis of the initial CT findings showing the duodenal-jejunal junction in the right hemi-abdomen, intestinal malrotation was suspected. However, because the jejunum deviated repeatedly to the right side but resolved spontaneously, we diagnosed dysplasia of the ligament of Treitz. Laparotomy revealed a formed ligament of Treitz; however, fixation in the upper jejunum was incomplete. Additionally, CT revealed that the anterior pararenal space was loosely fixed and mobile. These factors may have caused the right-sided deviation of the small intestine. In this case, the third part of the duodenum likely flexed on the right side of the aorta, causing an obstruction that resulted in repeat vomiting episodes and Mallory–Weiss syndrome.

## Clinical presentation

A 77-year-old woman with haematemesis presented to the emergency room. Her medical history included only hypertension and dyslipidaemia. When she presented to the emergency room, her vital signs indicated shock (heart rate: 100 beats/min, blood pressure: 79/56 mmHg), and blood tests revealed anaemia (haemoglobin: 9.6 g/dL), which suggested upper gastrointestinal bleeding.

**Figure 1.**
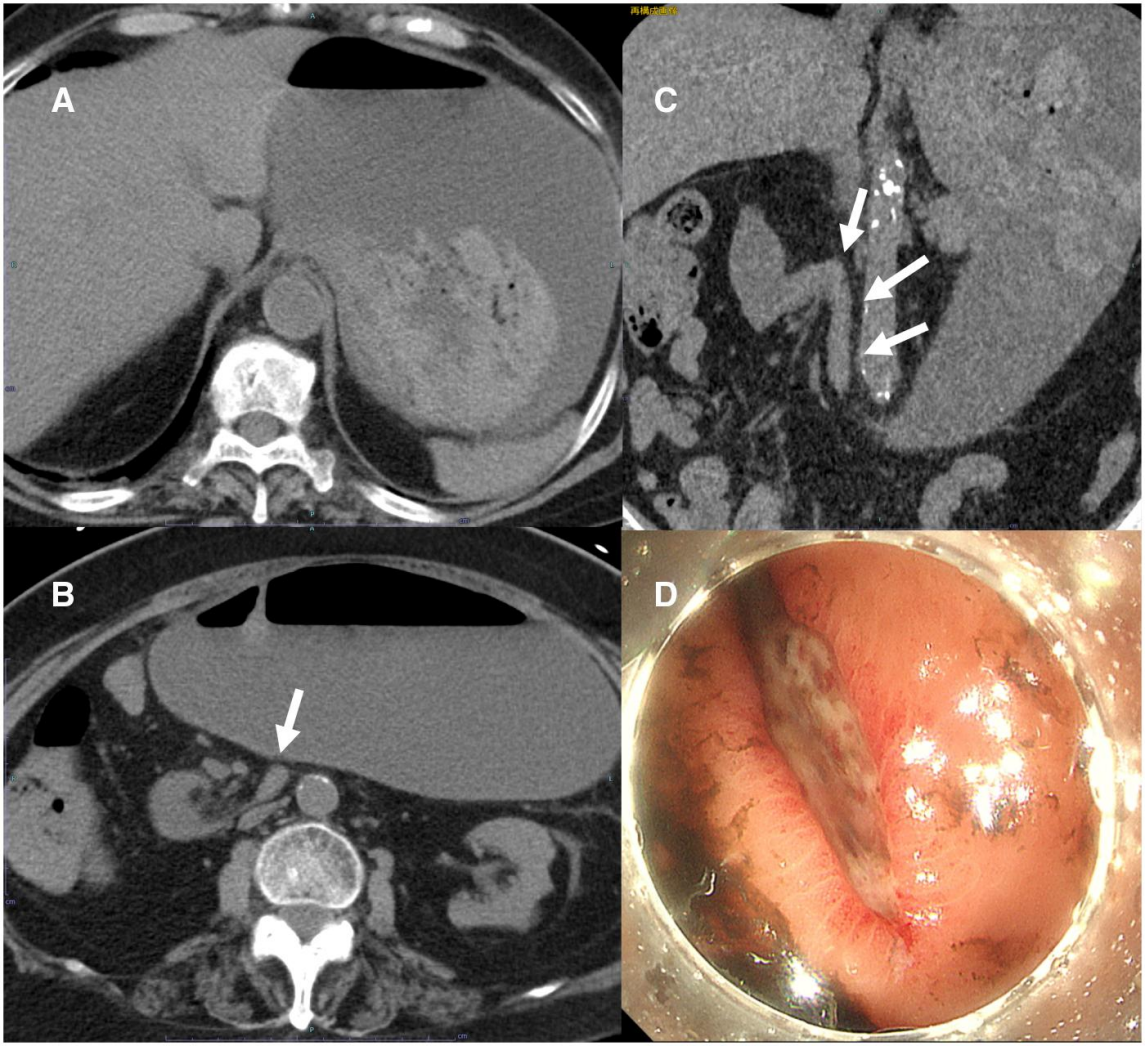
Axial non-contrast-enhanced CT images showing gastric dilatation and high-density contents that were considered a haematoma (A). Axial image (B) and coronal image (C) showing that the third part of the duodenum is flexed steeply on the right side of the aorta (arrow). Upper gastrointestinal endoscopy showing a laceration at the gastric cardia (D).

Non-contrast-enhanced CT was performed immediately because of renal dysfunction (Figure 1). CT revealed that the third part of the duodenum flexed steeply on the right side of the aorta and ran caudally, without crossing anterior to the aorta. The jejunum was located on the patient’s right side. The second part of the duodenum and the stomach were dilated, and there were high-density gastric contents that were considered to indicate a haematoma.

Upper gastrointestinal endoscopy was performed following the CT examination, which revealed a mucosal laceration at the gastric cardia. Bleeding from lacerations of the cardia of the stomach as a result of forceful vomiting was first reported by Mallory and Weiss in 1929.^1^ In our case, the third part of the duodenum flexed steeply, and the lumen was narrowed, which caused an obstruction. As a result, repeat vomiting was considered to have caused Mallory–Weiss syndrome.

**Figure 2.**
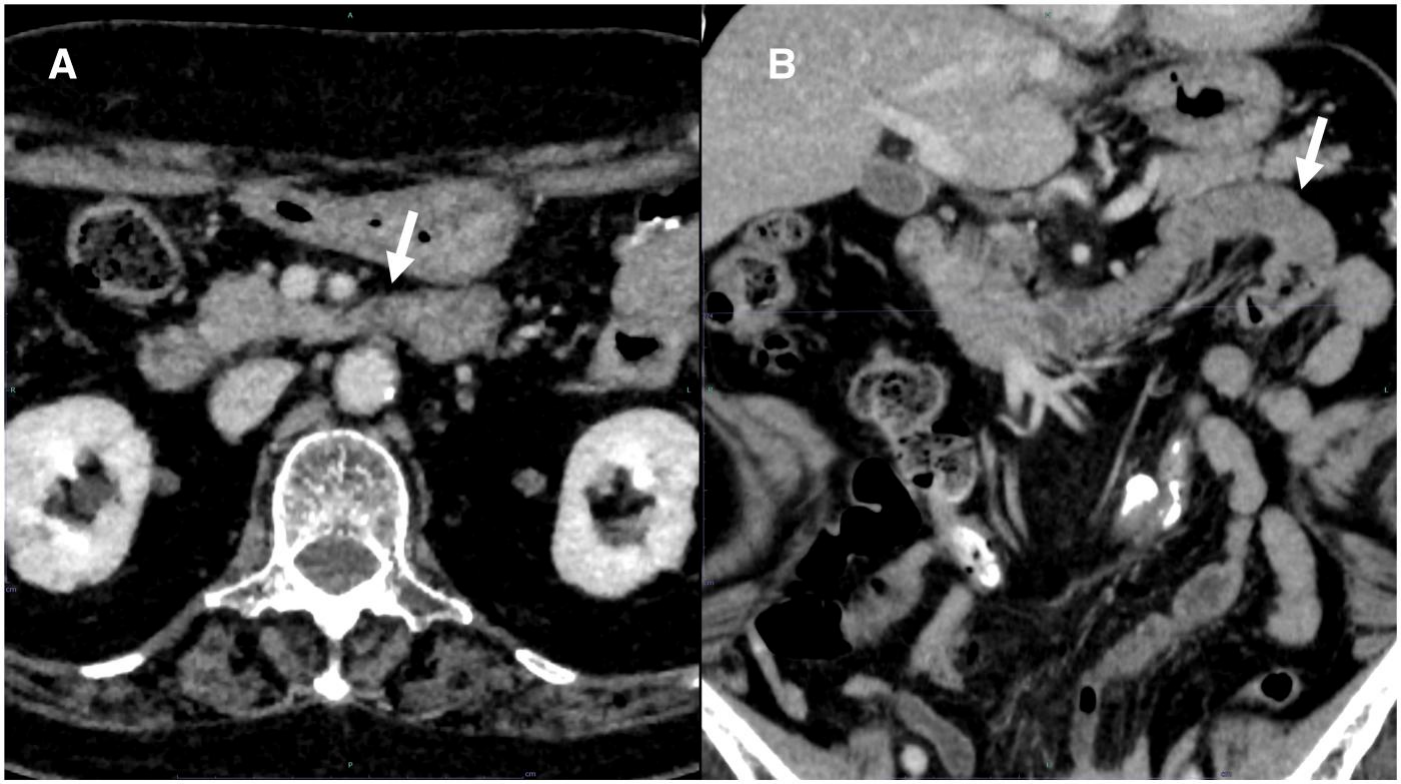
Contrast-enhanced CT obtained 7 days after the initial CT. The third part of the duodenum passes anterior to the aorta (A, arrow), and the jejunum is located on the patient’s left side (B, arrow), indicating spontaneous resolution of the previously right-sided duodenum and jejunum.

**Figure 3.**
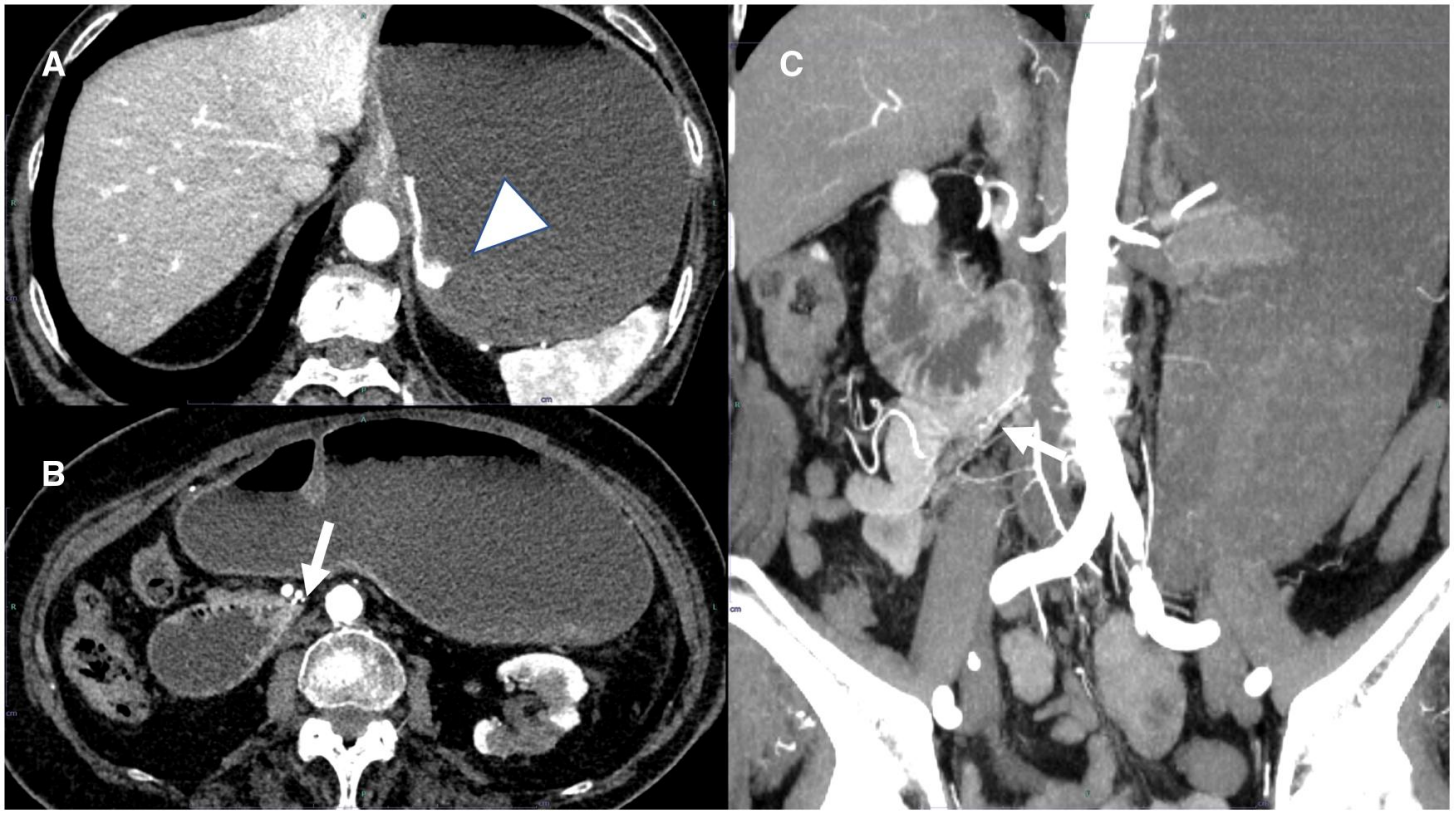
Dynamic CT performed 2 months after the first admission when the patient was rushed to the emergency room for repeat haematemesis. In the arterial phase, the stomach is dilated with fluid, and contrast extravasation from the gastric cardia to the gastric fundus is visible (A, arrowhead). The jejunum is also located on the right side (B, C: arrow).

On the basis of the CT findings showing that the duodenal-jejunal junction was located in the right hemi-abdomen, intestinal malrotation was suspected.^2^ However, 7 days later, when CT was repeated, spontaneous resolution of the malpositioned jejunum was seen (Figure 2). The patient was then discharged from the hospital. However, 2 months later, she was rushed to the emergency room for repeat haematemesis. Dynamic CT was performed before upper gastrointestinal endoscopy, on admission, and revealed contrast extravasation in the dilated stomach. Additionally, the third part of the duodenum was flexed on the right side of the aorta, and the duodenal-jejunal junction and jejunum were again located in the right hemi-abdomen (Figure 3). Upper gastrointestinal endoscopy revealed a laceration at the gastric cardia, as in the previous endoscopy, which was considered Mallory–Weiss syndrome.

**Figure 4.**
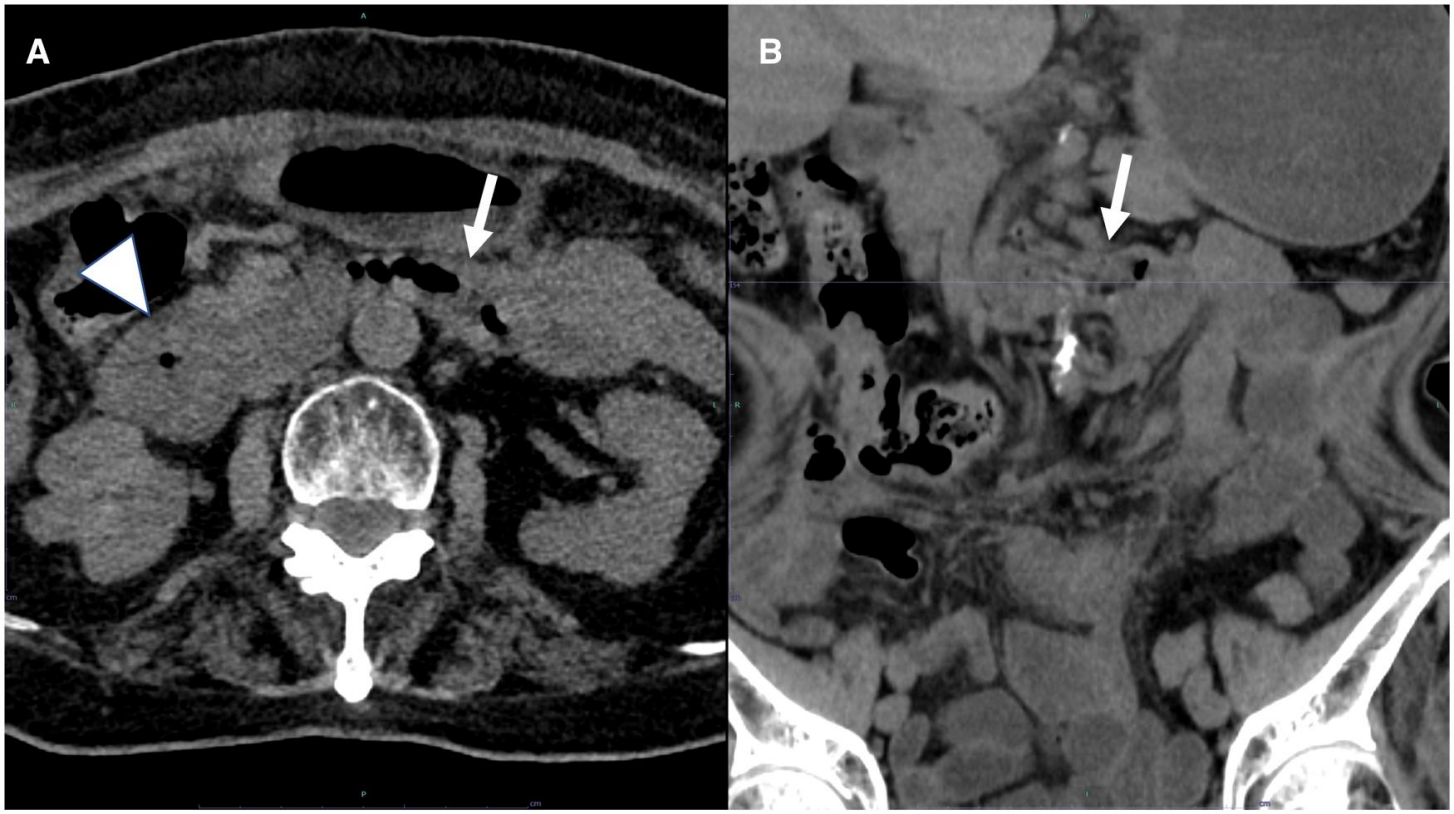
Two months after the second admission, non-contrast-enhanced CT was performed to evaluate nausea and abdominal pain (A, B). The CT images show no abnormalities in the position of the duodenum along its course (arrow). However, oedematous wall thickening is visible in the second part of the duodenum (A, arrowhead).

Two months after the second episode of haematemesis, the patient presented to the emergency room with nausea. Non-contrast-enhanced CT revealed no abnormalities in the duodenal positioning, but there was oedematous wall thickening in the second part of the duodenum (Figure 4). If we had not had previous CT images, we would have suspected duodenitis, but on the basis of all of the CT findings, we suspected the possibility of an underlying condition after the right-sided deviation of the small intestine had resolved spontaneously.

In summary, CT was performed 4 times over 5 months. The third and fourth parts of the duodenum and the jejunum deviated repeatedly, but this resolved spontaneously (Figure S1), which is not indicative of intestinal malrotation. Therefore, we diagnosed dysplasia of the ligament of Treitz.

## Clinical outcomes

The patient underwent laparotomy, which revealed no abnormalities in the relative position of the duodenum to the jejunum. Additionally, the jejunum was located on the patient’s left side, and there was no intestinal malrotation. The ligament of Treitz was formed; however, its fixation in the upper jejunum was incomplete as it was attached only to the duodenum. The duodenal-jejunal junction was not fixed to the retroperitoneum, and the jejunum folded easily with the ligament of Treitz as a fulcrum. Surgically, the upper jejunum was fixed with 4 sutures to the retroperitoneum on the patient’s left side. The postoperative course was good, and the patient has remained symptom-free.

## Discussion

Abnormal positioning of the duodenal-jejunal junction is one of the CT findings to consider in intestinal malrotation.^2^ In this case, the duodenal-jejunal junction was positioned abnormally during both episodes of haematemesis; however, repeat spontaneous resolution was observed. Therefore, we considered that the duodenal-jejunal junction and the upper jejunum were not fixed to the retroperitoneum around the ligament of Treitz.

The ligament of Treitz consists of 2 parts. The superior part is called the Hilfsmuskel and is a skeletal muscle from the right diaphragmatic crus at the oesophageal hiatus to the coeliac artery. The inferior part suspends the duodenum and the upper jejunum from the retroperitoneum.^3^ Unlike other ligaments in the abdomen, the ligament of Treitz has not been identified on CT and magnetic resonance imaging.^4^ This ligament shows considerable anatomic variations as it attaches to the distal attachment of the duodenum and the jejunum. The ligament commonly attaches to the third and fourth parts of the duodenum and the upper jejunum. The second common variation is that the ligament attaches to the third and fourth parts of the duodenum only, without an attachment to the upper jejunum. Attachment to the upper jejunum only, without duodenal attachment, is rare. There is also a type of multiple separate divisions and attachments to the duodenum.^2^ In our patient, the ligament was attached to the duodenum, but the upper jejunum was incompletely attached, as confirmed by surgery. Thus, the attachment was considered to correspond to the second common type of the variations of the ligament of Treitz.

**Figure 5.**
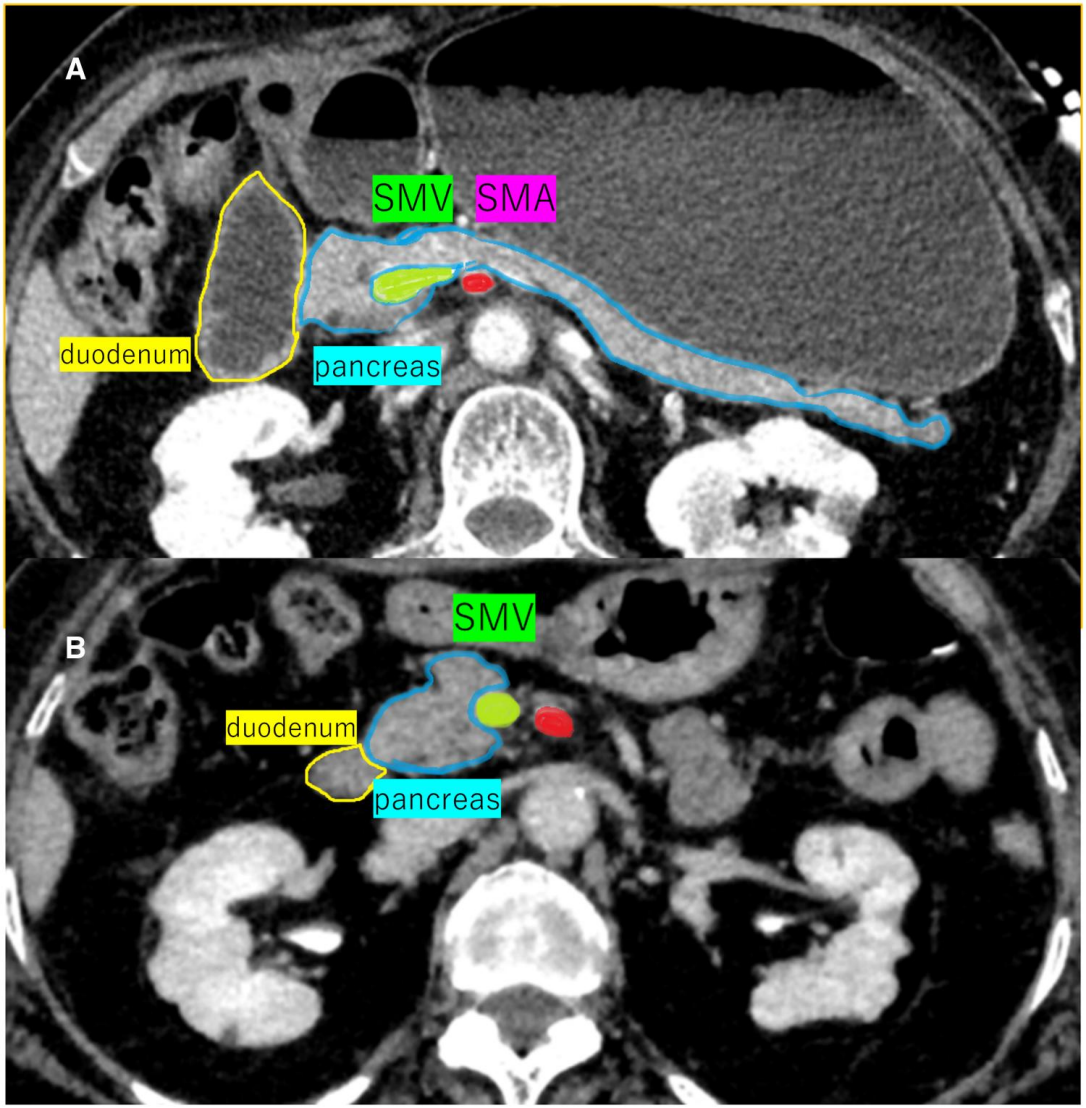
The upper CT image (axial view) is at the L2 level, and the image was obtained at the time of haematemesis. The image shows that the duodenal-jejunal junction is deviated to the right (A). The lower CT image (axial view) is at the same level and was obtained at the time of spontaneous resolution of the intestinal malpositioning (B). Comparing both CT images, the anterior pararenal space organs, such as the duodenum and pancreas, are shifted to the right when the position of the duodenal-jejunal junction is deviated to the right. Abbreviations: SMA = superior mesenteric artery; SMV = superior mesenteric vein.

The mobility of the anterior pararenal space in this case is important to discuss. The duodenum is the first segment of the small intestine and measures 25 cm in length. The duodenum is located mainly in the anterior pararenal space, in the retroperitoneum, except for the first half, which is intraperitoneal.^4^ Comparing our patient’s CT images of the duodenal-jejunal junction in abnormal positions with the images obtained at spontaneous resolutions, the organs of the anterior pararenal space, such as the duodenum and pancreas, were similarly shifted to the right when the position of the duodenal-jejunal junction was deviated to the right side (Figure 5).

Retroperitoneal organs, such as the pancreas and duodenum, are classically described as fixed and immobile.^5^ However, with the advent of imaging modalities, radiologists have frequently noticed mobility in the anterior pararenal space. For example, pancreatic mobility on respiration and changing body position has been reported.^5^^,^^6^ Morgan and Dubbins reported pancreatic mobility on changing position from supine to left posterior oblique positions. This mobility is more common in women than in men and may be associated with fragility of the retroperitoneal tissue due to ageing.^6^

Our patient had the anatomic variation of the ligament of Treitz of an unfixed upper jejunum. Additionally, the CT findings showed that the anterior pararenal space was loosely fixed and mobile. When rightward deviation of the anterior pararenal space occurred with positional change, the upper jejunum, which was not fixed, may have bent at the right side of the aorta. The steep flexure of the duodenum caused a narrowing of its lumen, and the oral intestinal tract became dilated, likely leading to repeat vomiting and subsequent Mallory–Weiss syndrome.

## Conclusion

We presented a case of small intestinal fixation failure. When right-sided deviation of the duodenal-jejunal junction is observed, intestinal malrotation is a differential. However, if repeat right-sided deviations occur and resolve spontaneously, this may involve anatomic variations in the ligament of Treitz and mobility of the anterior pararenal space.

## Learning points

- There are anatomic variations in the formation of the ligament of Treitz.
- Fixation of the organs of the anterior pararenal space may be loose, and the organs may shift with repositioning of the body and respiratory fluctuations.
- When CT reveals that the duodenal-jejunal junction is repeatedly deviated to the right and this resolves spontaneously, the underlying reason may be anatomic variations in the ligament of Treitz and mobility of the anterior pararenal space.

## Supplementary Material

uaae046_Supplementary_Data
