# Supplementary figures and images for: A case of small intestinal fixation failure
Source: BJR Case Rep. 2024 Nov 27;10(6):uaae046. doi: 10.1093/bjrcr/uaae046 (PMC11631180; doi:10.1093/bjrcr/uaae046)

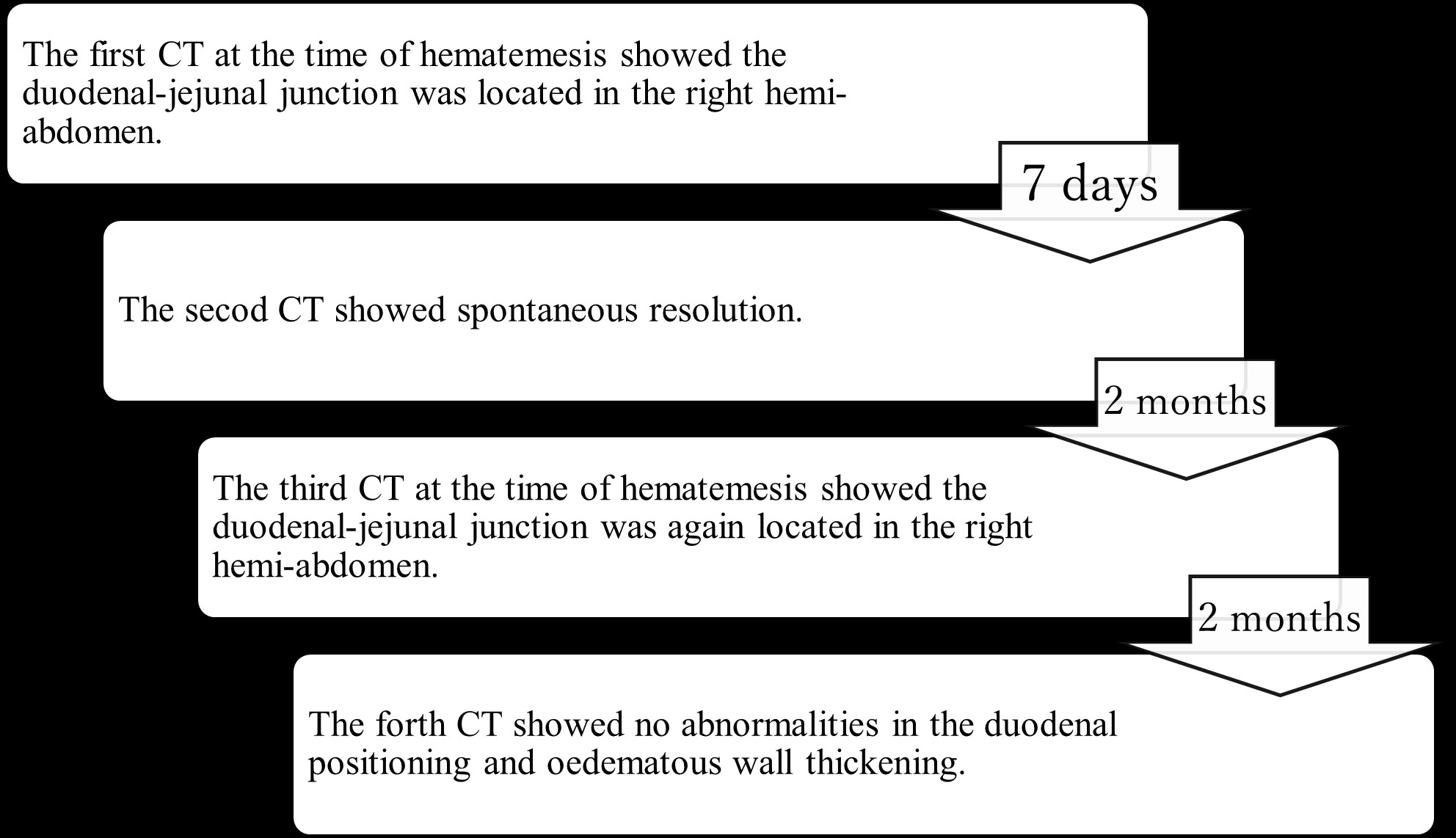

Supplement: uaae046_Supplementary_Data [file uaae046_supplementary_data.jpeg]
